# High-dose glucose–insulin–potassium has hemodynamic benefits and can improve cardiac remodeling in acute myocardial infarction treated with primary percutaneous coronary intervention: From a randomized controlled study

**DOI:** 10.4103/0975-3583.70899

**Published:** 2010

**Authors:** Yanhui Li, Lin Zhang, Lei Zhang, Haiyong Zhang, Nianzhong Zhang, Zhongsu Yang, Mingming Gao, Xinchun Yang, Liang Cui

**Affiliations:** *Heart Center, Beijing Chaoyang Hospital and Institute of Cardiovascular Disease, Capital Medical University, Beijing - 100020, P. R. China*

**Keywords:** Acute myocardial infarction, glucose–insulin–potassium, reperfusion therapy

## Abstract

**Objective:**

To evaluate the effects of high-dose glucose–insulin–potassium (GIK) solution on hemodynamics and cardiac remodeling in patients with acute myocardial infarction (AMI) treated with primary percutaneous coronary intervention (PCI).

**Patients and Methods:**

We observed the changes in the hemodynamic parameters in 26 patients with AMI. All patients received primary PCI before entering the study. All patients in the study were randomized into the GIK group (*n* = 14) or the control group (*n* = 12). Patients in the GIK group received high-dose GIK solution (25% glucose, 80 mmol/L KCl and 50 IU/L insulin; 1.5 ml/kg/h) over 24 h. Patients in the control group received standard therapy. We monitored the hemodynamic parameters at baseline and after 6 h, 12 h, 18 h and 24 h, respectively. Then, we followed-up the cardiac function with echocardiography after 7 days, 1 month and 6 months.

**Results:**

The basic clinical data was similar between the groups. Primary PCI was performed successfully in 25 patients. The two groups were indistinguishable in all factors measured. GIK solution did not have a deleterious effect on the hemodynamic parameters. The pulmonary capillary wedge pressure increased during the first 12-h period and then decreased smoothly (F = 3.75, *P* = 0.02). The trends were similar between the two groups. The system vascular resistance index (SVRI) and pulmonary vascular resistance index (PVRI) decreased during the first 12 h in the GIK group but increased in the control group. The GIK solution significantly influenced SVRI (F = 4.71, *P* = 0.02). GIK solution improved the cardiac function measured by stroke volume (F = 4.11, *P* = 0.03) and cardiac index (F = 4.40, *P* = 0.02). In the 6-month follow-up, GIK improved cardiac remodeling (left ventricular diastolic diameter: 49.2 ± 2.89 vs. 53.9 ± 2.48, *P* < 0.001; left ventricular systolic diameter: 32.9 ± 2.24 vs. 35.9 ± 2.78, *P* < 0.01).

**Conclusion:**

High-dose GIK solution had no adverse effects on the hemodynamics in AMI patients treated with primary PCI. It can improve cardiac function by lowering SVRI. In the 6-month follow-up, it improved cardiac remodeling.

## INTRODUCTION

The concept that the metabolic cocktail, glucose–insulin– potassium (GIK), may protect ischemic cardiomyocytes was first popularized by Sodi-Pallares as a “polarizing agent” in 1962.[1] Opie further proposed the rationale for the use of this metabolic therapy in 1970,[2] when he suggested two main mechanisms: the promotion of cardiac glycolysis and the diversion of free fatty acid (FFA) to adipocytes, with a resultant reduction in cardiac FFA metabolism.[3–9] In the “reperfusion therapy era,” the randomized controlled Estudios Cardiologicos Latinoamerica (ECLA) study suggested that only those patients who had received high-dose GIK therapy had a statistical survival advantage over the control group.[10–13] In another study, Ceremuzynski *et al*. did not show any beneficial effects of low-dose GIK therapy.[14] But, the acute hemodynamic effects and chronic cardiac function improvement of high-dose GIK therapy in acute myocardial infarction (AMI) patients were not well known, especially in patients treated with primary percutaneous coronary intervention (PCI).

The purpose of the present study is to analyze the hemodynamic changes during the first 24 h after intravenous administration of high-dose GIK solution in patients with a ST-segment elevated AMI who have received primary PCI.

## PATIENTS AND METHODS

The study was conducted as a consecutive-designed, randomized and blank-controlled study in our center.

The inclusion criteria were suspected symptoms or signs with typical electrocardiographic changes, which is ST-segment continuous elevation ≥1 mm in at least two adjacent leads or new LBBB(left bundle branch block), and time to be randomized ≤12 h from onset of symptoms.

The exclusion criteria were as follows:

1. Known renal impairment: the level of serum creatinine >2.0 mg/dl
2. Known hyperkalemia: the level of serum potassium >5.5 mmol/L

The purpose and details of the study were explained to all patients and written informed consent was obtained from all of them. The protocol was approved by the Ethical Committee of our hospital.

### Hemodynamic measurements

After admission, each subject had a chest X-ray and hemodynamic measurements were made under aseptic conditions in a special equipped room in the critical care unit. All patients underwent right-side heart catheterization at the bedside with a 4-lumen balloon-tipped thermodilution catheter (heparin-coated thermodilution catheter; 7Fr 4-lumin 110 cm; arrow AH-05000-H, Teleflex Incorporated, Limerick, PA, USA). The pressure was recorded by means of strain gauge transducers. The reference level was 5 cm below the sternum in the supine position. Cardiac output (CO) was determined with the thermodilution method. Each recorded value was based on a mean of at least two accepted measurements within 10% variation. All measurements and pressure curves were recorded on paper and saved for later analyses. After insertion of the catheters, the patients rested for 10 min and then a control reading, including CO, mean right atrial pressure (RAP), pulmonary artery pressure (PAP) and mean pulmonary capillary wedge pressure (PCWP), was performed. System blood pressure (BP) was obtained using a cuff-sphygmomanometer. Pressure monitoring and hemodynamic parameter calculating were completed using the Eagle 4000 patient monitor instruction (GE Medical Systems Company, Marquette, WI, USA). The following indices were derived from the measurement obtained. Cardiac index (CI) = CO/body surface area (BSA), stroke volume (SV) = CO/heart rate (HR) *1000, mean artery pressure (MAP) = (SBP-DBP)/3 + DBP, system vascular resistance (SVR) = (MAP-RAP) * 79.92/ CO, system vascular resistance index (SVRI) = SVR/BSA, pulmonary vascular resistance (PVR) = (PAP-PCWP) * 79.92/CO, pulmonary vascular resistance index (PVRI) = PVR/BSA, left ventricular stroke work index (LVSWI) = SV * (MAP-PCWP) * 0.0136/BSA, right ventricular stroke work index (RVSWI) = SV * (PAM-RAP) * 0.0136/BSA. Hemodynamic measurements were repeated at baseline, 6 h, 12 h, 18 h and 24 h. The BSA was calculated based on the formula, BSA = height (m)^0.725^ * weight (kg)^0.425^ * 0.007184.

## DRUG ADMINISTRATION

Preparation of high-dose GIK solution (glucose 25%, insulin 50 IU/L and KCl 80 mmol/L): 10% glucose 300 ml + 50% glucose 200 ml + 15% KCl 20 ml + insulin 26 IU.

If the patient was randomized into the GIK group, GIK solution was given by an infusion of 1.5 ml/kg/h over 24 h after baseline hemodynamic measurement obtained. If the patient was randomized into the control group, standard therapy was given.

### Correction of the speed of GIK infusion

The initial dose of GIK is standard. The speed of the GIK infusion was not adjusted before the serum concentration of glucose and potassium were obtained. When the serum glucose concentration was between 250 mg/dl and 350 mg/dl before or during the intravenous administration, it was considered to be down-regulated and then the patients were administered extra insulin by means of intravenous or hypodermal routes rather than adjusting the GIK infusion. However, in the presence of following conditions: pulmonary edema, serum glucose > 350 mg/ dl in the 6^th^ hour after GIK infusion or serum potassium > 5.5 mmol/L, GIK infusion speed was adjusted.

### Biochemical analysis

Laboratory analyses including serum concentration of glucose and potassium before randomization (baseline), 6 h and 24 h after randomization and the levels of serum cTnI, were performed. The serum concentrations of glucose and potassium were measured with a fully automated biochemical instrument. The levels of serum cTnI were analyzed using the OPUS instrument.

### Statistical methods

All continuous data were expressed as mean ± standard deviation. Repeated-measures ANOVA was used to assess the effects of intervention factor (GIK infusion) on hemodynamics. Time was as repeated-measurement factors. The type I error was kept at 5% (α = 0.05). A *P* value of <0.05 was considered statistically significant.

## RESULTS

### Patient characteristics [Table 1]

**Table 1:** Patient characteristics

Sex	Age	Group	MI location	IRA	No. of lesion artery	cTnI
M	75	1	Infra-posterior, RV	RCA	2	35.7
M	44	2	Extensive anterior	LAD	1	29.9
M	77	1	Anterior	LAD	2	0.133
M	70	1	Extensive anterior	LAD	2	>50
F	70	1	Anterior	LAD	3	13
M	71	2	Extensive anterior	LAD	2	>50
M	68	2	Inferior, RV	RCA	3	0.601
M	80	1	Inferior, RV	RCA	3+ LM	>50
M	58	2	Infra-posteriorlateral	LCX	3	0.424
F	70	2	Extensive anterior	LAD	3	>50
F	64	1	Inferior	RCA	1	9.07
F	64	2	Inferior, RV	RCA	2	3.63
M	64	2	Inferior	RCA	3+ LM	21.2
F	64	2	Infra-posterior	RCA	3	>50
M	50	1	Infra-posteriorlateral	LCX	2	7.07
F	67	2	Anterior	LAD	3	14.1
M	67	1	Infra-posterior, RV	RCA	3	28.5
M	59	2	Infra-posterior	RCA	3	>50
F	68	2	Inferior, RV	RCA	3+ LM	>50
M	77	1	Infra-posterior	RCA	2	6.28
F	63	1	Anterior	LAD	3+ LM	>50
M	66	1	Anterior	LAD	2	7.68
F	68	1	Inferior, RV	RCA	2	>50
M	61	2	Infra-posteriorlateral	LCX	3	11.7
F	71	1	Inferior, RV	RCA	3+LM	26.4
M	40	1	Anterior	LAD	1	>50

M, male; F, female; Group 1, GIK group; Group 2, control group; IRA, infarctionrelated vessel; LM, left main; RCA, right coronary artery; LAD, left anterior descending; LCX, left circumflex

Primary PCI was performed in 25 patients. One additional patient did not receive myocardial reperfusion therapy.

### Clinical findings

The tendency of HR and BP is shown in Table 2. There are no significant differences between the two groups [Table 2]. Also, the high-dose GIK infusion has no significant effect on the HR and BP (HR: F = 0.46, *P* = 0.67; MAP: F = 0.93, *P* = 0.43). The serum concentrations of glucose and potassium increased significantly due to GIK infusion [Tables 3 and 4]. Generally, the serum glucose concentration in patients with diabetes mellitus (DM) was easy to be increased, yet it could be controlled to a satisfactory level with the adjustment of insulin administration. Although the potassium level increased, it was still in the normal range (<5.0 mmol/L). The GIK was never stopped or decelerated because of the progress of pulmonary edema. All the patients recovered and were discharged. During the hospitalization period, no malignant arrhythmia, reinfarction, heart failure, recurrent ischemia, hypoglycemia, cardiogenic shock or cardiac arrest occurred. Among the subjects, selected CABG (coronary artery bypass graft) was performed in three cases during the hospitalization period. Two patients in the GIK group experienced diuresis, about 4000–5000 ml/d. They were given proper fluid for the maintenance of blood volume and electrolytes.

**Table 2:** The HR and BP at baseline, 6 h, 12 h, 18 h and 24 h in the two groups

	GIK group (*n* = 14)					Control group (*n* = 12)				
	Baseline	6 h	12 h	18 h	24 h	Baseline	6 h	12 h	18 h	24 h
HR (bpm)	71 ± 14	71 ± 13	73 ± 15	72 ± 13	74 ± 11	71 ± 9	70 ± 9	68 ± 10	70 ± 9	72 ± 10
SBP (mmHg)	126 ± 16	125 ± 15	122 ± 24	118 ± 21	116 ± 12	118 ± 20	118 ± 16	123 ± 17	121 ± 15	120 ± 14
DBP (mmHg)	73 ± 7	71 ± 5	69 ± 7	72 ± 8	69 ± 7	70 ± 12	71 ± 9	71 ± 8	70 ± 10	69 ± 8
MAP (mmHg)	91 ± 7	89 ± 7	86 ± 11	87 ± 12	85 ± 6	86 ± 14	87 ± 11	88 ± 10	87 ± 11	86 ± 9

**Table 3:** Blood glucose and serum potassium levels before and after GIK infusion

	GIK group (*n* = 14)			Control group (*n* = 12)		
	Baseline	12 h	24 h	Baseline	12 h	24 h
cTnI (ng/ml)	25 ± 21			29 ± 21		
Serum glucose (mg/dl)	157 ± 37	2 48 ± 99**	170 ± 95	22 2 ± 10 3*	188 ± 77	148 ± 57
Serum potassium (mmol/L)	3.9 ± 0.6	4.2 ± 0.7	4.6 ± 0.6	4.3 ± 0.5	4.2 ± 0.2	4.1 ± 0.3#

* P <0.05,

** P <0.05 vs. GIK baseline;

# P <0.05 vs. GIK 24 h

**Table 4:** Statistical results of serum glucose and potassium

	Serum potassium		Serum glucose	
Effects	F-value	*P* value	F-value	*P* value
Time	2.818	0.073	6.170	0.008
Time*group	7.173	0.002	7.178	0.004
Group	0.026	0.873	0.035	0.854

### Acute hemodynamic effects

Among the 26 subjects, the Swan-Ganz catheter could be introduced into the pulmonary artery under pressure wave form monitoring at the bedside in 25 patients. In other 1 patient, it’s failed under pressure wave form monitoring at the bedside, then successful with underlying X-ray in catheter lab. Pressure curves and thermodilution curves of good quality were registered in all patients during the entire period. The hemodynamic data are summarized in Table 5 and Table 6. Soon after AMI, RAP increased slightly and then decreased gradually 12 h later (F = 2.2, *P* = 0.12). But, there were no significant differences on the RAP between the GIK and the control groups (F = 0.06, *P* = 0.81). Highdose GIK infusion had no significant effect on the RAP (F = 0.15, *P* = 0.87). Similar to RAP, the PCWP also increased soon after AMI, then decreased 6 h later (F = 3.75, *P* = 0.02). However, compared with the control, high-dose GIK infusion decreased PCWP soon after AMI. In the control group, the SVRI increased from 2706 ± 817 to 2952 ± 392 after 12 h. But, it decreased from 2859 ± 531 to 2322 ± 436 in the GIK group. The SVRI decreased gradually after 24 h in the two groups. High-dose GIK infusion had a significant effect on the SVRI (F = 4.71, *P* = 0.02). The PVRI in the control group increased slightly at an earlier time and that of the GIK group presented a decreasing trend. There was no significant effect of different times on the PVRI (F = 0.19, *P* = 0.81). High-dose GIK infusion had no statistically significant effect on the PVRI (F = 1.57, *P* = 0.22). The LVSWI increased gradually from 35 to 39 within 24 h after AMI (F = 1.91, *P* = 0.17). High-dose GIK infusion increased the LVSWI slightly, without any statistical significance (F = 1.55, *P* = 0.23). The changes of RVSWI have the same tendency as LVSWI. SV showed an increasing trend from 60.2 ± 16.5 ml to 68.9 ± 23.6 ml in the GIK group, yet that in the control showed a decreasing trend from 60.0 ± 17.7 ml to 54.8 ± 11.0 ml at first and then increased gradually 24 h later. High-dose GIK infusion increased the SV significantly (F = 4.11, *P* = 0.03). Similar to SV, CI showed an increasing trend from 2.4 ± 0.5 to 2.8 ± 0.8 in the GIK group, yet that of the control presented a decreasing trend from 2.5 ± 0.6 to 2.2 ± 0.4 within 12 h after AMI. High-dose GIK infusion improved the CI significantly (F = 4.39, *P* = 0.02). There were no significant differences in SV and CI between the GIK group and the control 24 h later.

**Table 5:** Hemodynamic data at different times between the two groups

	GIK group (*n* = 14)					Control group (*n* = 12)				
	Baseline	6 h	12 h	18 h	24 h	Baseline	6 h	12 h	18 h	24 h
RAP	7.9 ± 4.5	8.3 ± 3.5	8.3 ± 3.5	7.6 ± 4.3	6.3 ± 4.0	6.9 ± 5.4	8.4 ± 4.6	7.9 ± 3.9	7.1 ± 2.0	6.3 ± 2.3
PCWP	12.3 ± 2.8	15.0 ± 4.8	12.8 ± 4.7	10.3 ± 6.2	9.8 ± 4.6	12.2 ± 4.8	13.2 ± 4.8	11.8 ± 3.6	12.5 ± 5.3	10.4 ± 5.3
SVRI	2859 ± 531		2322 ± 436		2308 ± 315	2706 ± 817		2952 ± 392***		2494 ± 666
PVRI	277 ± 125		245 ± 98		215 ± 104	206 ± 89		259 ± 112		250 ± 95
LVSWI	35.6 ± 10.1		39.3 ± 13.5		38.7 ± 11.0	35.4 ± 12.1		34.0 ± 8.4		39.0 ± 9.6
RVSWI	5.6 ± 2.1		6.3 ± 2.3		5.7 ± 2.7	5.9 ± 4.0		5.0 ± 2.6		6.4 ± 3.3
SV	60.2 ± 16.5		68.9 ± 23.6		68.0 ± 21.9	60.0 ± 17.2		54.8 ± 11.0		63.7 ± 14.4
CI	2.4 ± 0.5		2.8 ± 0.8		2.7 ± 0.4	2.5 ± 0.6		2.2 ± 0.4		2.7 ± 0.6

*** P <0.001 vs. GIK 12 h

**Table 6:** Results of statistical analysis on hemodynamics

	Time		Time*group		Group	
	F-value	*P* value	F-value	*P* value	F-value	*P* value
RAP	2.197	0.120	0.154	0.873	0.062	0.807
PAWP	3.754	0.019	1.042	0.378	0.000	0.986
SVRI	4.522	0.020	4.706	0.017	1.182	0.291
PVRI	0.186	0.814	1.574	0.222	0.071	0.793
LVSWI	1.907	0.168	1.545	0.229	0.156	0.698
RVSWI	0.213	0.774	1.401	0.259	0.008	0.931
SV	3.076	0.061	4.108	0.027	0.785	0.307
CI	3.293	0.051	4.393	0.022	0.766	0.392

### Improvement of cardiac remodeling

**Figure 1:**
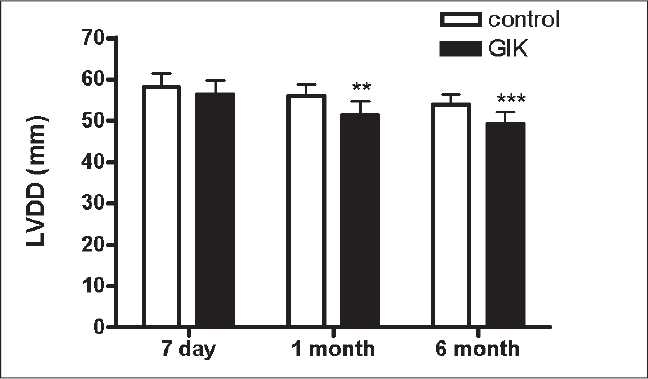
The changes of left ventricular diastolic diameter from day 7 to 6 months between the glucose–insulin–potassium group and the control group **P<0.01 vs. GIK, ***P<0.001 vs. GIK

**Figure 2:**
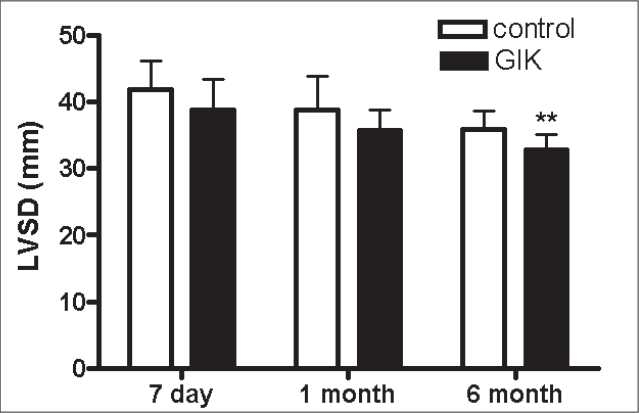
The changes of left ventricular systolic diameter from day 7 to 6 months between the glucose–insulin–potassium group and the control group **P<0.01 vs. GIK

**Figure 3:**
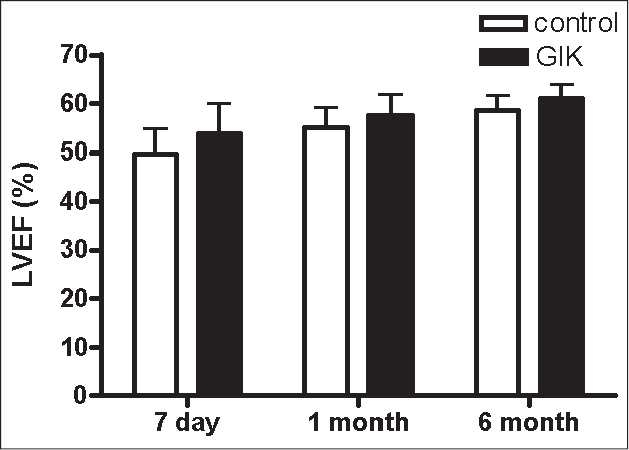
The changes of LVEF from day 7 to 6 months between the glucose–insulin–potassium group and the control group

After the treatment, during the acute period, all patients received standard therapy. A 6-month follow-up was performed. We assessed cardiac function and cardiac remodeling by echocardiography after 7 days, 1 month and 6 months. We observed that left ventricular diastolic diameter (LVDD) and left ventricular systolic diameter (LVSD) decreased from 7 days to 6 months in all patients. There were significant differences in LVSD between the groups after the 6-month treatment. There were significant differences in LVDD between the groups after the 1- and 6-month treatment [Figures 1–3].

### Adverse effects

Among the 26 subjects, two cases demonstrated hyperglycemia, although the hyperglycemia was corrected after hypodermal insulin administration and deceleration of GIK. No hyperkalemia was recorded. No patients in the GIK group manifested acute heart failure during the high-dose GIK infusion. The high-dose GIK infusion in acute MI patients is safe.

## DISCUSSION

Our study has clearly shown that early after AMI, highdose GIK infusion improves cardiac function, as judged by hemodynamic measurements. Previous studies have shown the potential beneficial effects of GIK on the reduction of infarct size, improvement in global ejection fraction in infarcted zone, lower pulmonary pressures and decrease in diastolic and systolic volumes.[15] The clinical beneficial effects of high-dose GIK infusion and its mechanism are not, however, fully understood. Our previous study had shown that high-dose GIK can decrease the cardiomyocyte apoptosis in AMI patients with reperfusion therapy.[16] With high-dose GIK infusion, CI was increased and SV was improved without an increase in HR in our study. This suggests that GIK infusion can improve cardiac function in AMI patients at an early phase,[3,4,6,17,18] and that increase in the reflex HR is not the mechanism. Our results showed that SVRI increased early after AMI. However, SVRI decreased significantly in patients treated with high-dose GIK and increase in LVSWI did not reach statistical significance. This indicates that SVRI lowering may play a key role in the mechanism. Because LVSWI increased slightly (difference not significant), GIK solution can protect cardiomyocytes to some degree, although a large-scale international randomized trial, the CREATE-ECLA trial,[19] indicated that high-dose GIK infusion had a neutral effect on mortality, cardiac arrest and cardiogenic shock in patients with acute STEMI during a 30-day follow-up. In the CREATE-ECLA trial, not all patients received primary PCI. And, data from the CREATE trial is not similar to that from the ECLA trial. In our clinical practice, we found that high-dose GIK infusion could improve the clinical conditions in patients with cardiogenic shock due to AMI.

Also, we found that LVDD, LVSD and LVEF (left ventricular ejection fraction) improved after the 6-month standard therapy in all patients. Interesting, this was more significant in the GIK group, suggesting that GIK could improve cardiac remodeling in AMI patients receiving primary PCI. But, the mechanism is not well known. Some mechanisms have been proposed for the association of GIK solution therapy with improved cardiac remodeling, including decreasing cardiomyocyte apoptosis, improving myocardial perfusion, improving myocardial metabolism, lowering SVRI and so on.

In conclusion, the preliminary observation suggests that high-dose GIK infusion results in improved cardiac function in the “myocardial reperfusion era” and SVR lowering could play some role in this mechanism.

## LIMITATION

As the sample size is small in the study, it could potentially bias the results of the evaluation. If the sample size is large enough, increase in the LVSWI could reach statistical significance. If this were the case, high-dose GIK infusion could improve myocardial contractility in some way.
